# Impact on clinical outcome of ventricular arrhythmias in patients undergoing transcatheter aortic valve implantation

**DOI:** 10.2459/JCM.0000000000001596

**Published:** 2024-02-12

**Authors:** Nello Cambise, Eleonora Gnan, Saverio Tremamunno, Alessandro Telesca, Antonietta Belmusto, Lorenzo Tinti, Antonio Di Renzo, Cristina Aurigemma, Francesco Burzotta, Carlo Trani, Filippo Crea, Gaetano Antonio Lanza

**Affiliations:** a Università Catto1ica del Sacro Cuore; b Fondazione Policlinico Universitario ‘Agostino Gemelli’ IRCCS, Department of Cardiovascular Sciences, Rome, Italy

**Keywords:** clinical outcome, ECG Holter monitoring, transcatheter aortic valve implantation, ventricular arrhythmias

## Abstract

**Background:**

Transcatheter aortic valve implantation (TAVI) has become a largely used treatment for severe aortic stenosis. There are limited data, however, about predictors of long-term prognosis in this population. In this study, we assessed whether ventricular arrhythmias may predict clinical outcomes in patients undergoing TAVI.

**Methods and results:**

We performed a 24 h ECG Holter monitoring in 267 patients who underwent TAVI for severe aortic stenosis within 30 days from a successful procedure. The occurrence of frequent premature ventricular complexes (PVCs; ≥30/h), polymorphic PVCs and nonsustained ventricular tachycardia (NSVT) was obtained for each patient. Clinical outcome was obtained for 228 patients (85%), for an average follow-up of 3.5 years (range 1.0–8.6). Cardiovascular events (CVEs; cardiovascular death or resuscitated cardiac arrest) occurred in 26 patients (11.4%) and 63 patients died (27.6%). Frequent PVCs but not polymorphic PVCs and NSVT were found to be associated with CVEs at univariate analysis. Frequent PVCs were indeed found in 12 patients with (46.2%) and 35 without (17.3%) CVEs [hazard ratio 2.30; 95% confidence interval (CI) 1.03–5.09; *P* = 0.04], whereas polymorphic PVCs were found in 11 (42.3%) and 54 (26.7%) patients of the two groups, respectively (hazard ratio 1.44; 95% CI 0.64–3.25; *P* = 0.38), and NSVT in 9 (34.6%) and 43 patients of the two groups, respectively (hazard ratio 1.18; 95% CI 0.48–2.87; *P* = 0.72). Frequent PVCs, however, were not significantly associated with CVEs at multivariate Cox regression analysis (hazard ratio 1.53; 95% CI 0.37–6.30; *P* = 0.56). Both frequent PVCs, polymorphic PVCs and NSVT showed no significant association with mortality.

**Conclusion:**

In our study, the detection of frequent PVCs at Holter monitoring after TAVI was a predictor of CVEs (cardiovascular death/cardiac arrest), but this association was lost in multivariable analysis.

## Introduction

Severe aortic stenosis is associated with a significant increase in mortality and morbidity, with prognosis substantially deteriorating as soon as typical symptoms appear.^1,2^ An increased burden of ventricular arrhythmias has been reported in these patients.^3,4^ The presence of frequent and/or complex ventricular arrhythmias in severe aortic stenosis is likely the result of multiple factors, including left ventricle hypertrophy, increased myocardial work, subendocardial ischemia, myocardial fibrosis and left ventricle dysfunction. Importantly, as in patients with coronary artery disease or cardiomyopathy, high-grade ventricular arrhythmias might be associated with increased risk of cardiovascular events, in particular sudden death, which has been reported to be the cause of death in a sizeable proportion of patients with symptomatic aortic stenosis.^3,4^

Transcatheter aortic valve implantation (TAVI) has become a standard treatment for elderly patients with severe aortic stenosis. Clinical outcome in patients treated by TAVI is influenced by several factors, including the presence of atrial fibrillation, moderate-to-severe mitral regurgitation, use of proton-pump inhibitors^5^ and comorbidities, as well as frailty.^5–9^

Whether the detection of ventricular arrhythmias has any prognostic implications in these patients, however, has poorly been investigated.^10–13^

## Methods

### Patients

We prospectively enrolled patients admitted to our Department of Cardiovascular Sciences of the University Hospital Agostino Gemelli IRCCS, Rome, Italy, between 2013 and 2021, who underwent TAVI for severe aortic stenosis, defined as Doppler aortic valve area (D-AVA) less than 1.0 cm^2^ (or indexed D-AVA <0.60 cm^2^/m^2^), resulting in typical symptoms, including dyspnoea, syncope or presyncope and angina.

Patients were excluded in case of: presence of other severe valvular heart disease, any clinically relevant congenital heart disease, a previous known history of cardiomyopathy, or an acute coronary syndrome in the previous 12 months; indication to TAVI for failing surgical aortic bioprostheses or for severe aortic insufficiency; severe acute complications related to TAVI, defined as any procedure-related complications that required surgical/interventional procedures (other than pacemaker implantation) or that caused death during hospitalization; refusal of patients to participate in the study; technical issues with Holter recordings that make arrhythmia analysis unreliable.

The protocol was approved by our Institutional Review Board. All enrolled patients were informed of the nature and purpose of the study and gave written informed consent for their participation.

### Study protocol

Clinical data were acquired for each patient, including cardiovascular risk factors, i.e., hypertension (blood pressure ≥140/90 mmHg and/or intake of antihypertensive medication), hypercholesterolemia (total cholesterol levels ≥200 mg/dl and/or LDL cholesterol ≥130 mg/dl and/or intake of lipid-lowering drugs), diabetes mellitus (glycated haemoglobin serum concentration ≥6.5% and/or fasting blood glucose ≥126 mg/dl and/or use of antidiabetic drugs). Furthermore, frailty of patients was indicated by a clinical frailty scale score of 6 or higher.^14^

Prescribed medications at discharge and presence of any comorbidity were also recorded, with particular attention to the presence of chronic kidney disease (defined as a baseline glomerular filtration rate <60 ml/min/1.73 m^2^), coronary artery disease (i.e. history of previous acute myocardial infarction and coronary revascularization by percutaneous coronary intervention or coronary artery bypass graft surgery) and peripheral arterial disease (PAD; defined as presence of significant stenosis in lower limb arteries diagnosed by echocardiographic-colour-Doppler or contrast CT scan).

### Transthoracic Doppler echocardiography

A standard transthoracic echocardiogram (TT-ECHO) was performed before TAVI and before discharge after TAVI, using a Philips EPIQ Ultrasound System (Philips, Milan, Italy).

The echocardiographic assessment of aortic valve stenosis was made according to EACVI-ASE 2017 recommendations^15^ and included the calculation of transvalvular pressure gradients, Doppler Velocity Index and Stroke Volume index. The mean transvalvular gradient was calculated from the average of the instantaneous gradients recorded during systole, derived from the contour of the Doppler velocity curve.

D-AVA was calculated using the Doppler method with a continuity equation. Left ventricle ejection fraction (LVEF) and left atrium volume index (LAVi) were calculated by the Simpson biplane method. Left ventricle mass was also obtained applying the Devereux formula^16^ and indexed by body surface area. Systolic pulmonary artery pressure (sPAP) was calculated as the sum of the tricuspid insufficiency peak gradient and the estimated right atrial pressure. We also considered the presence of moderate valvular regurgitations, according to 2017 ASE Guidelines.^17^

Paravalvular leak (PVL) was defined as the presence of one or more paravalvular leaks of the implanted aortic valve determining an at least moderate valve insufficiency.

### Twenty-four-hour electrocardiogram Holter monitoring

All patients underwent a 24 h ECG Holter monitoring (HM-ECG) within 30 days from TAVI using three-channel recorders (Schiller Medilog AR4) and monitoring the bipolar chest leads CM5, CM1 and modified aVF. HM-ECG recordings were analysed by an experienced operator using the system Medilog Darwin 2.1 (Schiller Medilog, Baar, Switzerland).

The presence and characteristics of ventricular arrhythmias at HM-ECG were assessed. Specifically, we aimed to identify and assess the prognostic value of frequent premature ventricular complexes (PVCs; defined as 30 or more PVCs per hour), polymorphic PVCs and episodes of nonsustained ventricular tachycardia (NSVT; defined as the presence of three or more consecutive PVCs with a rate ≥100 bpm).

### Follow-up

The clinical status of patients was evaluated by telephone calls or programmed medical examination. The primary endpoint of the study was a composite of cardiovascular mortality and resuscitation from cardiac arrest (primary cardiovascular events; CVEs). Total mortality was also assessed as a unique secondary endpoint.

### Statistical analysis

Data for continuous variables are reported as means ± standard deviation, whereas discrete variables are reported as numbers and percentages. The association of variables with the endpoints was assessed by univariate Cox regression analysis. Independent predictors of outcomes were identified by multivariable Cox regression analysis, including variables with *P*-value less than 0.1 at univariate assessment. Survival curves of patients with or without frequent PVCs were constructed with the Kaplan–Meier method and compared by log-rank test. A *P* less than 0.05 was always required for statistical significance. Statistical analyses were performed with the SPSS 28.0 software (SPSS Italia, Florence, Italy).

## Results

### Characteristics of patients and outcome

Overall, we enrolled 267 consecutive patients who successfully underwent TAVI for severe aortic stenosis and fulfilled the inclusion exclusion criteria. Clinical outcome could be obtained in 228 patients (85%), who constituted the final population of the study. The population included 96 male individuals (42.1%) and 171 female individuals (57.8%) and the mean age was 81.1 ± 7.3 years.

During a mean follow-up period of 3.5 years (range 1–8.6), CVEs occurred in 26 (11.4%) patients, consisting of cardiovascular death in 24 and resuscitation from cardiac arrest in 2, whereas 63 patients (27.6%) died. Thus, cardiovascular and noncardiovascular mortality in our population was 10.5 and 17.1%, respectively.

### Predictors of cardiovascular events

Table 1 shows the main clinical characteristics of patients with or without CVEs. Hypercholesterolemia and diabetes were the only clinical findings associated with the primary endpoint. Hypercholesterolemia was, in fact, associated with a lower risk of CVEs [hazard ratio 0.11; 95% confidence interval (CI) 0.27–0.69; *P* = 0.006], while diabetes was instead associated with a higher risk of CVEs (hazard ratio 2.27; 95% CI 1.01–5.10; *P* = 0.047).

**Table 1:** Relationship of clinical data of patients with cardiovascular events at follow-up

	CVEs (*n* = 26)	NO CVEs (*n* = 202)	Crude HR (95% CI)	*P*
Age (years)	78.6 ± 8.4	81.5 ± 7.1	0.97 (0.93–1.01)	0.09
Male	17 (65.4%)	79 (39.1%)	2.01 (0.91–4.77)	0.08
Hypertension	22 (84.6%)	164 (81.2%)	0.87 (0.29–2.59)	0.80
Hypercholesterolemia	6 (23.1%)	110 (54.5%)	0.11 (0.27–0.69)	0.006
Diabetes	11 (42.3%)	47 (23.3%)	2.27 (1.01–5.10)	0.047
Obesity	5 (19.2%)	27 (13.4%)	2.42 (0.90–6.52)	0.08
Chronic kidney disease	1 (3.8%)	26 (12.9%)	0.34 (0.05–2.52)	0.29
Previous AMI	1 (3.8%)	8 (4%)	1.33 (0.18–10.0)	0.78
Previous PCI/CABG	5 (19.2%)	56 (27.7%)	0.62 (0.23–1.66)	0.34
PAD	2 (7.7%)	6 (3%)	1.33 (0.30–5.19)	0.71
ST-T changes	17 (65.4%)	113 (55.9%)	0.88 (0.36–2.18)	0.78
Frailty	17 (65.4%)	118 (58.4%)	1.18 (0.51–2.77)	0.69
AF history	39 (19.3%)	6 (23.1%)	0.95 (0.35–2.55)	0.91
New-onset AF	0 (0%)	16 (7.8%)	0.05 (0.00–214.42)	0.48

AF, atrial fibrillation; AMI, acute myocardial infarction; CABG, coronary artery bypass graft surgery; CI, confidence interval; CVEs, cardiovascular events; HR, hazard ratio; PAD, peripheral arterial disease; PCI, percutaneous coronary intervention.

Table 2 summarizes the relationship of the main echocardiographic findings with CVEs. Preprocedural D-AVA (hazard ratio 20.2; 95% CI 2.08–197.3; *P* = 0.01) and paravalvular leak determining moderate/severe aortic valve insufficiency (hazard ratio 2.34; 95% CI 1.05–5.22; *P* = 0.038) were found to be predictive of the primary endpoint, as was the postprocedural sPAP value (hazard ratio 1.05; 95% CI 1.05–1.08; *P* = 0.003).

**Table 2:** Relationship of echocardiographic data before and after transcatheter aortic valve implantation with cardiovascular events at follow-up

	CVEs (*n* = 26)	No CVEs (*n* = 202)	Crude HR (95% CI)	*P*
Data pre-TAVI
AVA-D (cm^2^)	0.71 ± 0.21	0.67 ± 0.16	20.2 (2.08–197.3)	0.01
Aortic valve mean gradient (mmHg)	52.6 ± 17.0	53.5 ± 15.4	0.97 (0.95–1.00)	0.06
LVEF (%)	53.8 ± 15	56.6 ± 12	0.98 (0.95–1.01)	0.15
LVMi (g/m^2^)	154 ± 37	141 ± 42	1.00 (0.99–1.01)	0.65
LAVi (ml/m^2^)	56.7 ± 23	49.9 ± 17	1.01 (0.99–1.03)	0.44
Aortic valve insufficiency	12 (46.2%)	39 (20.2%)	2.34 (1.05–5.22)	0.038
Mitral valve insufficiency	8 (30.8%)	57 (29.5%)	1.03 (0.51–2.06)	0.94
Tricuspid valve insufficiency	7 (26.9%)	39 (20.3%)	1.47 (0.59 – 3.67)	0.41
sPAP (mmHg)	47.1 ± 18	40.3 ± 12	1.02 (0.97–1.05)	0.10
Data post-TAVI
Aortic valve mean gradient (mmHg)	10.8 ± 6.6	10.0 ± 6.7	0.98 (0.92–1.03)	0.40
LVEF (%)	55.7 ± 12	57.2 ± 11	0.99 (0.95–1.03)	0.47
LVMi (g/m^2^)	151.4 ± 41	137.9 ± 38	1.01 (0.99 (1.02)	0.34
LAVi (ml/m^2^)	45.2 ± 18	49.8 ± 19	0.99 (0.98–1.01)	0.49
Aortic valve insufficiency/PVL	3 (12%)	17 (8.8%)	1.71 (0.47–6.17)	0.42
Mitral valve insufficiency	3 (12.5%)	16 (8.2%)	1.65 (0.48–5.65)	0.43
Tricuspid valve insufficiency	4 (16%)	24 (12.9%)	1.57 (0.53–4.64)	0.42
sPAP (mmHg)	43.9 ± 17	35.9 ± 12	1.05 (1.02–1.08)	0.003

AVA-D, aortic valve area–Doppler; CI, confidence interval; CVEs, cardiovascular events; HR, hazard ratio; LVEF, left ventricular ejection fraction; LVMi, left ventricular mass index; LAVi, left atrium volume index; PVL, paravalvular leak; sPAP, systolic pulmonary arterial pressure.

### Ventricular arrhythmias and cardiovascular events

**Fig. 1:**
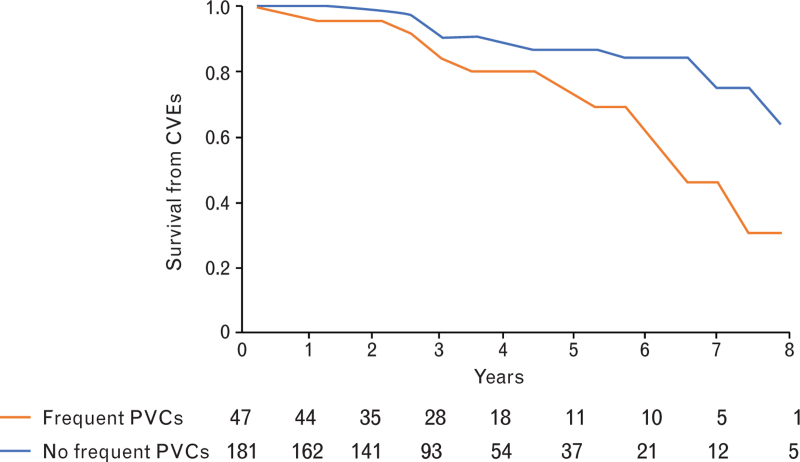
Kaplan–Meier survival curves from cardiovascular events in transcatheter aortic valve implantation patients with or without frequent premature ventricular complexes at 24 h ECG Holter monitoring (unadjusted comparison by log-rank test).

The relationship of ventricular arrhythmias with CVEs is shown in Table 3. Frequent PVCs (≥30/h), but not polymorphic PVCs and NSVT were significantly associated with CVEs. Frequent PVCs (≥30/h) were present in 12 patients with (46.2%) and 35 patients without (17.3%) CVEs (hazard ratio 2.30; 95% CI 1.04–5.09; *P* = 0.04). The survival curves of patients with or without frequent PVCs, and the statistical result of their comparison by log-rank test, are shown in Fig. 1 (central figure).

**Table 3:** Ventricular arrhythmias in patients with or without cardiovascular events

	CVEs (*n* = 26)	NO CVEs (*n* = 202)	Crude HR (95% CI)	*P*
Frequent PVCs (≥30/h)	12 (46.2%)	35 (17.3%)	2.30 (1.04–5.09)	0.041
Polymorphic PVCs	11 (42.3)	54 (26.7%)	1.44 (0.64–3.25)	0.38
NSVT	9 (34.6%)	43 (21.3%)	1.18 (0.48–2.87)	0.72
Frequent and polymorphic PVCs	9 (34.6%)	15 (7.4%)	2.53 (1.07–6.00)	0.035
Frequent PVCs and NSVT	1 (3.8%)	12 (5.9%)	0.52 (0.07–3.89)	0.52

CI, confidence interval; CVEs, cardiovascular events; HR, hazard ratio; NSVT, nonsustained ventricular tachycardia; PVCs, premature ventricular complexes.

On the other hand, polymorphic PVCs were detected in 11 (42.3%) and 54 patients (26.7%) with and without CVEs, respectively (hazard ratio 2.53; 95% CI 1.07–6.00; *P* = 0.035) at univariate analysis, and NSVT episodes in 9 patients with (34.6%) and 43 patients without (21.3%) CVEs, respectively (hazard ratio 1.18; 95% CI 0.48–2.87; *P* = 0.72).

At multivariable analysis, however, frequent PVCs lost their association with CVEs (hazard ratio 1.59; 95% CI 0.39–6.45; *P* = 0.51), whereas only hypercholesterolemia was found to be (inversely) independently associated with CVE (hazard ratio 0.16; 95% CI 0.03–0.88; *P* = 0.035; Table 4).

**Table 4:** Results of multivariable Cox regression analysis for cardiovascular events

	HR (95% CI)	*P*
Age	0.99 (0.93–1.06)	0.82
Male	1.33 (0.34–5.18)	0.68
Hypercholesterolemia	0.16 (0.03–0.88)	0.035
Diabetes	1.51 (0.38–6.01)	0.56
Obesity	5.18 (0.64–42.18)	0.12
Pre-TAVI AVA-D	1.25 (0.01–129.89)	0.93
Pre-TAVI AV mean gradient (mmHg)	0.98 (0.93–1.02)	0.29
Pre-TAVI aortic insufficiency	1.42 (0.37–5.42)	0.61
Post-TAVI sPAP (mmHg)	1.04 (0.99–1.09)	0.07
Frequent PVCs (>30/h)	1.59 (0.39–6.45)	0.51
Frequent polymorphic PVCs (>30/h)^a^	1.42 (0.32–6.35)	0.65

AVA-D, aortic valve area–Doppler; AV, aortic valve; LVEF, left ventricle ejection fraction; sPAP, systolic pulmonary artery pressure; PVCs, premature ventricular complexes.

a Included in alternative to frequent PVCs (the risk associated with the other variables remained substantially unchanged).

Considering the presence of both frequent and polymorphic PVCs together only slightly and nonsignificantly increased the risk of CVEs (Table 3) and was also not associated with CVEs at multivariable analysis (Table 4). Considering both the presence of frequent PVCs and NSVT, on the other hand, showed no significant association with CVEs, likely because of the low number of patients with this finding (Table 3).

### All-cause mortality

No clinical variable was significantly associated with all-cause mortality at univariate Cox regression analysis, but several pre-TAVI and/or post-TAVI echocardiographic findings were associated with mortality, including D-AVA, aortic valve mean gradient, LVEF, pre-TAVI mitral insufficiency and post-TAVI sPAP (Table 5).

**Table 5:** Relationship with all-cause mortality of clinical data and echocardiographic findings before and after transcatheter aortic valve implantation

Clinical characteristics					
	HR (95% CI)	*P*		HR (95% CI)	*P*
Age (years)	1.00 (0.97–1.03)	0.95	Previous AMI	0.55 (0.08–3.98)	0.55
Male	1.33 (0.80–2.21)	0.27	PAD	0.82 (0.25–2.67)	0.74
Hypertension	1.15 (0.54–2.44)	0.71	Previous PCI/CABG	0.81 (0.46–1.44)	0.47
Hypercholesterolemia	0.74 (0.44–1.23)	0.24	ST-T changes	0.93 (0.52–1.64)	0.80
Diabetes	1.03 (0.58–1.81)	0.92	Frailty	0.79 (0.47–1.34)	0.39
Obesity	1.94 (0.97–3.85)	0.06	AF history	1.12 (0.62–2.05)	0.71
Chronic kidney disease	0.78 (0.33–1.82)	0.56	New-onset AF	1.18 (0.37–3.79)	0.78

Echocardiographic findings				
	Pre-TAVI		Post-TAVI	
	HR (95% CI)	*P*	HR (95% CI)	*P*
AVA-D	4.83 (0.99–23.7)	0.052	–	–
Aortic valve mean gradient	0.98 (0.97–0.99)	0.041	0.97 (0.94–1.01)	0.12
LVEF	0.98 (0.96–0.99)	0.013	0.98 (0.96–1.01)	0.22
LVMi	0.99 (0.99–1.00)	0.46	1.00 (0.99–1.00)	0.86
LAVi	1.00 (0.99–1.02)	0.56	1.00 (0.99–1.01)	0.47
Aortic valve insufficiency	1.46 (0.84–2.52)	0.18	1.32 (0.55–3.17)^a^	0.53
Mitral valve insufficiency	1.47 (1.04–2.07)	0.029	1.94 (0.91–4.14)	0.087
Tricuspid valve insufficiency	1.64 (0.93–2.89)	0.086	1.66 (0.83–3.3)	0.15
sPAP (mmHg)	1.02 (0.99–1.04)	0.058	1.03 (1.01–1.03)	0.16

a Paravalvular leak with at least moderate aortic valve insufficiency. AMI, acute myocardial infarction; AVA-D, aortic valve area–Doppler; CABG, coronary artery bypass graft surgery; CI, confidence interval; CVEs, cardiovascular events; HR, hazard ratio; LVEF, left ventricular ejection fraction; LVMi, left ventricular mass index; LAVi, left atrium volume index; PVL, paravalvular leak; sPAP, systolic pulmonary arterial pressure.

Ventricular arrhythmias were not found to be significantly associated with total mortality. Frequent PVCs were detected in 19 patients (30.2%) who died and 44 patients (17.0%) who survived during follow-up (hazard ratio 1.20; 95% CI 0.69–2.08; *P* = 0.53); polymorphic PVCs in 16 patients (25.4%) who died and 49 patients (29.7%) who survived (hazard ratio 0.85; 95% CI 0.41–1.74; *P* = 0.65); and NSVT in 18 patients who died (28.6%) and 34 patients (20.6%) who survived (hazard ratio 0.93; 95% CI 0.52–1.65; *P* = 0.79).

No clinical or echographic variable, however, was found to be an independent predictor of death at multivariable analysis.

## Discussion

To the best of our knowledge, this is the first study that aimed to assess the prognostic role of ventricular arrhythmias in patients with severe aortic stenosis treated by TAVI. Our data show that the detection of frequent PVCs at HM-ECG performed in the following 30 days after TAVI was associated with a worse cardiovascular outcome.

Previous studies showed that ventricular arrhythmias are frequent in patients undergoing TAVI and decrease in incidence and severity during follow-up after the first month,^4,10,13^ but no previous study investigated the prognostic implications of frequent or complex ventricular arrhythmias in these patients.

In our study, the detection of frequent PVCs at HM-ECG in patients who underwent TAVI was associated with an increased rate of CVEs. The association, however, was lost after adjustment for significant clinical and echocardiographic variables. This finding may not be surprising. Sudden death is a well known cause of death in patients with aortic valve stenosis, and ventricular arrhythmias have been hypothesized to be responsible for the high incidence of sudden death in symptomatic patients with aortic stenosis,^3,18^ although this association has never clearly been demonstrated. The presence of ventricular arrhythmias might represent a marker of severity of myocardial involvement, consisting of structural abnormalities (i.e. left ventricular hypertrophy and fibrosis) as well as secondary pulmonary hypertension, consequent to persistently elevated afterload caused by aortic stenosis.^3,18^ These abnormalities may persist despite the removal of left ventricle afterload with TAVI and may be responsible for negative clinical outcomes.

It is interesting to observe that we did not find any significant association between echocardiographic findings, related to the severity of aortic stenosis and its effects on LV dynamics (e.g. trans-aortic mean gradient, LVMi, LAVi, LVEF), and CVEs, as well as with all-cause mortality. Only hypercholesterolemia maintained statistical significance at multivariate analysis, resulting in an inverse association with CVEs. This apparently surprising finding might be explained, at least in part, by the use of adequate statin therapy in patients with increased levels of blood cholesterol^19^ or merely related to chance. However, the association of reduced cholesterol levels with clinical outcome in TAVI was also found in other studies,^20^ and therefore deserves further future investigation.

On the whole, our results are in agreement with previous studies, which showed conflicting data regarding the predictive value for clinical events of general and echocardiographic variables in patients undergoing TAVI.

Previous studies have reported varying and inconsistent associations between cardiovascular events, mortality, and both clinical factors (such as age,^6,8^ male sex,^8,21^ presence of coronary artery disease,^22^ diabetes,^23^ chronic kidney disease,^8,20^ and smoking) and echocardiographic factors (including low LVEF,^7,24,25^ mean AV gradient,^7,26^ PAPs,^7,9,23^ and moderate-to-severe mitral^23^ and aortic regurgitation^7^).

Finally, it should be observed that, at variance with most data in medical literature,^19,21–26^ in our population, we recorded a higher rate (17.1%) of noncardiovascular than cardiovascular (10.5%) mortality. The reasons for this finding are not clear, but the inclusion of more generally compromised patients, as indicated by the high rate of ‘frail’ patients (59.6%), may at least in part explain the unusual high rate of noncardiovascular death.

### Limitations of the study

Some limitations of our study should be acknowledged. First, the sample size was relatively small and might have been insufficient to confirm an independent association of frequent PVCs with the cardiovascular outcome. Thus, our results suggest that larger studies deserve to be conducted to better analyse the relationship between ventricular arrhythmias and clinical outcome. Second, our follow-up was limited to 3.5 years and, therefore, whether ventricular arrhythmias might predict clinical events over a longer-term period (e.g. 5 years) deserves further investigations.

## Conclusion

Our data show that the detection of frequent PVCs at HM after TAVI, performed for severe aortic stenosis, was a predictor of CVEs (cardiovascular death/cardiac arrest), but this association was lost in multivariable analysis. Our data, however, suggest that further larger studies deserve to be conducted to better clarify the prognostic role of ventricular arrhythmias in TAVI patients.

### Conflicts of interest

There are no conflicts of interest.
